# Biochemical Changes in Human Cells Exposed to Low Concentrations of Gold Nanoparticles Detected by Raman Microspectroscopy

**DOI:** 10.3390/s19102418

**Published:** 2019-05-27

**Authors:** Maria Lasalvia, Giuseppe Perna, Vito Capozzi

**Affiliations:** Dipartimento di Medicina Clinica e Sperimentale, Università di Foggia, 71122 Foggia, Italy; maria.lasalvia@unifg.it (M.L.); vito.capozzi@unifg.it (V.C.)

**Keywords:** Raman microspectroscopy, human cells, nanotoxicology

## Abstract

The toxicological implications of nanoparticles deserve accurate scientific investigation for the protection of human health. Although toxic effects involve specific organs, the events that cause them have their origin from biochemical modifications of some cellular constituents. Therefore, a first analysis to evaluate the effects due to the action of nanoparticles is achieved by investigation of in vitro cells, which allows the identification of the cellular modifications caused by nanoparticles (NPs) even at much lower doses than the lethal ones. This work evaluated the Raman microspectroscopy capability to monitor biochemical changes occurring in human cells as a consequence of exposure to a suspension of gold nanoparticles with a non-cytotoxic concentration. Human keratinocyte cells were used as a model cell line, because they are mainly involved in environmental exposure. A trypan blue assay revealed that the investigated concentration, 650 ng/mL, is non-cytotoxic (about 5% of cells died after 48 h exposure). Specific Raman spectral markers to represent the cell response to nanoparticle exposure were found (at 1450 and 2865 cm^−1^) in the cytoplasm spectra, with the aid of ratiometric and principal component analysis.

## 1. Introduction

Over the past few decades, gold nanoparticles (NPs) have been largely manufactured and investigated by considering possible biomedical applications, such as drug delivery [1], imaging [2], photothermal therapy [3], photodynamic therapy [4], cancer diagnosis and therapy [5], and so forth, because it is known that gold is an inert and biocompatible material.

Although NP-based techniques have led to the achievement of technological progress in all fields of application, their use might have a significant impact on health, due to their extremely small size and very high surface/volume ratio, that makes them highly reactive [6]. This characteristic can cause their toxicity when they come into contact with biological systems. Among metal NPs, the gold-based ones are considered inert and biocompatible materials. Recently, in vitro studies have shown that cell exposure to gold NPs produces dose- and time-dependent toxicity expressions on different cell lines [7,8,9], although such an issue is still largely debated [10,11].

Conventional in vitro cyto-toxicological techniques can produce valuable information about particle toxicity. In particular, the trypan blue test can specify the eventual NP-induced damage of the plasmatic membrane, an MTT assay can produce information about the role of NPs in the alteration of mitochondrial activity, and crystal violet analysis is able to evaluate NP-induced changes of the DNA amount in exposed cells. The results of such cytological tests are considered as an estimate of cellular viability, because normal cells preserve the integrity of plasmatic membrane and they keep unchanged the mitochondrial activity and DNA production. Also, histochemical methods can provide proper tools for detecting and quantitatively analyzing different cellular components by means of dye staining and using suitable fluorescent antibodies. However, colorimetric assays have some restrictions about the possibility of application. In fact, the MTT viability assay, measured by means of yellow light absorbance, the crystal violet assay, which estimates the absorption of green light, and intracellular reactive oxygen species production, investigated by means of blue excitation and green fluorescence emission, can yield unreliable results when applied to gold NP-exposed samples, because of NP absorption in the green-yellow spectral range. In addition, each cytotoxicity assay measures the toxic response of cells in different ways, and the relative sensitivity of the assay depends on the assay sensitivity to events involved in the toxic action. Therefore, the achievement of information about many cellular compartments by means of a single assay based on the analysis of multiple parameters is still a notable challenge in nanotoxicology. 

Raman microspectroscopy represents a suitable tool to provide such multiple biochemical information about toxicant action. In fact, Raman microspectroscopy is a vibrational technique capable of providing information about the biochemical composition of functional groups inside single cells by analyzing the spectral composition of the light diffused by them after exposure to an appropriate laser beam frequency. Since any pathological condition corresponds to an alteration of the biochemical content and/or the cellular structures, such changes are reflected in the Raman spectral data sets and can be used to identify different cellular events [12]. In fact, in recent years this spectroscopy technique has attracted the attention of the medical community as a new non-invasive tool for in vitro analysis of tissues and biological fluids [13]. In particular, several works have demonstrated the potential of Raman spectroscopy to provide a diagnostic tool that will enable clinicians to identify early neoplastic changes [14,15,16].

Recently, Efeoglu et al. [17,18,19] showed the ability of Raman spectroscopy to provide information about cellular responses at different concentrations of aminated polystyrene (PS-NH_2_) nanoparticles and exposure times with the aid of multivariate analysis. In particular, they identified specific spectral markers reflecting changes in nucleic acid, protein, and lipid components for different cell lines exposed to such NPs at sub-lethal and lethal doses. However, PS-NH_2_ NPs have been considered as a positive cytotoxicity model for many cell lines [20]. Usually, cytotoxicity events appear after exposure to a sufficiently large NP dose, as a consequence of interaction between NPs and cellular constituents, which are irreversibly damaged by the chemical action of the toxicity. The chemical action of NPs occurs also at low doses, although they are not sufficient to kill a large number of cells. Nonetheless, in vitro exposure to a low dose of NPs can give indications about the proper dose at which exposure is still tolerable by the cells. Therefore, it is interesting to investigate the capability of Raman microspectroscopy to detect biochemical modifications occurring in cells exposed to a low dose of gold NPs, which are inert and biocompatible. Further, it is worth evaluating the use of Raman microspectroscopy also as a biosensing technique to monitor cellular responses to NP systems. 

In this study, the biochemical effects due to 24 and 48 h exposure of human cells to a low concentration of gold NPs have been monitored using Raman microspectroscopy, with the aim of identifying some specific spectral markers of early toxic action. The investigated concentration value was clearly non-cytotoxic, as confirmed by the trypan blue assay. Gold NPs were chosen because they are widely used in biomedical applications. Human keratinocyte cells were chosen as the investigated sample because they can act as models for human exposure to environmental nanomaterials. Specific Raman spectral markers, which represent the cell response to gold NP exposure, have been found to be the lipid-related bands (at 1450 and 2865 cm^−1^) in the cytoplasm, with the aid of ratiometric and principal component analysis.

## 2. Materials and Methods

### 2.1. Cell Culture and NP Exposure

Normal human keratinocyte cells (HuKe cell line, IZSLER, Brescia, Italy) were grown in Epilife medium, supplied with Keratinocyte Medium Supplement, at 37 °C, 5% CO_2_. One day before exposure, they were seeded in poly-lysine coated glass coverslips, located inside six-well plates, at densities of 7 × 10^3^ cells for each well and grown for 24 h.

Commercially available 40 nm diameter gold NPs, stabilized in 0.1 mM phosphate buffered saline (PBS) suspension at a concentration ~7.2 × 10^10^ NPs/mL (purchased from Sigma-Aldrich, Milano, Italy) were chosen as model nanoparticles for this study. The nanoparticles were prepared in cell culture medium on the day of exposure, with a NP-suspension/medium volume ratio of about 1/30.

Therefore, the samples for Raman microspectroscopy and cytotoxicity assays consisted of cultured cells on glass coverslips, exposed to a gold NP suspension with a concentration of about 650 ng/mL, corresponding to about 10^6^ NPs/cell. After 24 h and 48 h exposure, control and exposed cell samples were washed three times in phosphate buffered saline (PBS) and then fixed in paraformaldehyde 3.7%. Cells to be analyzed by Raman microspectroscopy were fixed in order to prevent morphological and chemical changes during data acquisition. Then, the samples were stored in Petri dishes filled with PBS solution until Raman analysis. 

### 2.2. Cell Viability Assay

The trypan blue method was used to evaluate the toxicity of NP solution for HuKe cells. This method estimates cell viability by means of plasma membrane integrity. Cells with a damaged cell membrane (necrotic cells) stain blue, whereas cells with an intact plasma membrane (healthy or apoptotic cells) remain unstained. Cells were harvested with trypsin and a small aliquot of the cell suspension was diluted with an equal volume of 0.4% trypan blue solution (Sigma-Aldrich). Cells were then counted under a phase-contrast microscope.

### 2.3. Raman Spectroscopy

Raman spectra were measured at room temperature by means of a Raman confocal micro-spectrometer apparatus (LabRAM from Jobin-Yvon Horiba, Milano, Italy), as described elsewhere [21]. Briefly, the 514.5 nm line of an Ar ion laser, 10 mW power, was focused by an 100× oil-immersion objective (Olympus, Tokyo, Japan, numeric aperture = 1.4) to obtain a diffraction-limited spot of about 0.5 µm diameter, that was used to excite two different locations of single HuKe cells, i.e., a cell volume including the nucleus and a cell volume excluding it. The Raman spectra were measured, in the 700–1800 (low wavenumber) and 2700–3150 cm^−1^ (high wavenumber) spectral ranges, for about 30 randomly chosen single cells for each type of cellular sample (control and NP exposed), with an acquisition time of 10 s for each single measured spectrum and the signal was averaged over three acquisitions. The light scattered from the sample was collected in backscattering geometry and passed through an edge filter (Omega Optical, Inc., Brattleboro, VT, USA) to suppress the laser line. Then, light crossed a square confocal hole (300 µm diagonal) and a 200 µm entrance slit of a spectrometer equipped with a 600 grooves/mm grating. The signal was detected by means of a cooled charge-coupled device (CCD). The spectral resolution was ~5 cm^−1^/pixel. Raman spectra of the background signal (the coated glass coverslip and the PBS solution) were acquired by moving the objective, without varying the focus position, to a nearby region where there were no cells.

### 2.4. Spectral Processing and Data Analysis

Each Raman spectrum was analyzed by subtracting firstly the corresponding background signal and, successively, the cell fluorescence and stray light signal (by means of an automated baseline removal method included in the LabSpec software, Jobin-Yvon Horiba). Then, the obtained spectrum was normalized, by dividing each wavenumber intensity value by the total spectral area, in order to minimize the effect of fluctuation either in the sampling of the cellular volume or in the laser power on the spectral intensity of each single cell. Finally, the normalized spectra of each cell sample were independently averaged over the total number of measured spectra to obtain an average spectrum for both the control and exposed cells. 

Ratiometric analysis was performed by calculating the intensity ratio of some specific couples of Raman peaks for each normalized single spectrum. Successively, the mean value and standard error were calculated for each set of values of the investigated intensity ratios, related to the exposed and control sample and measured for the two cellular locations. The statistical difference between the exposed group and the control group was assessed by the Holm–Sidak method. P values < 0.05 were considered to be statistically significant. All statistical analyses were performed by SigmaPlot software (version 12.5, Systat Software). Principal component analysis (PCA) was performed by means of the ChemoSpec package in R software [22]. 

## 3. Results and Discussion

### 3.1. Optical Characterization of NPs

The incubation of gold NPs in cell medium is a fundamental step of the in vitro exposure experiments. Therefore, the effects of the medium to the NPs have been tested by means of optical absorption measurements in the visible range (400–800 nm), i.e., absorption spectra of a cuvette containing a suspension of gold NPs inside culture medium have been performed. These optical spectra have been carried out with a UV-visible spectrophotometer (Lambda 40, Perkin Elmer, Waltham, MA, USA). In fact, visible radiation is selectively absorbed, according to the NP size, as a result of excitation of surface plasmons [23]. As the frequency of absorbed radiation depends on the NP size (it increases with decreasing NP size), the spectral position of the absorption peak can estimate the average size of investigated NPs [24]. 

**Figure 1:**
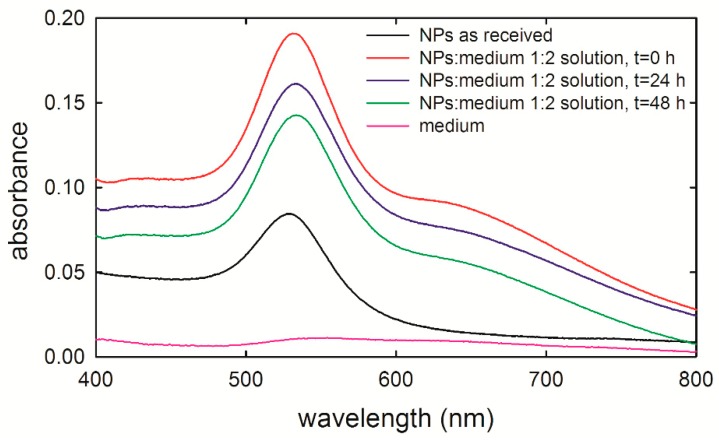
Absorbance spectra of 40 nm gold nanoparticles (NPs) stabilized in 0.1 mM phosphate buffered saline (PBS), measured as received (black line) and by suspending them in the cell medium, at the beginning of the incubation time (red line), after 24 h (blue line), and 48 h (green line) of incubation. The NPs-PBS suspension/medium volume ratio is 1:2. The absorption spectrum of cell medium is also shown (pink line). The spectra have been shifted on the vertical axis for clarity.

Figure 1 shows the absorption spectra of the as-received NP suspension (dark line) and three different suspensions of NPs in the cellular medium measured immediately (red line), 24 h (blue line), and 48 h (green line) after preparation. Such optical measurements were carried out with a NP concentration quite higher than that used for exposure experiments—indeed, the measured sample consisted of one part of NPs inside PBS and two parts of medium, because the NP concentration used for exposure experiments (NP-suspension/medium volume ratio of about 1/30) did not result in a measured signal resolved with respect to the background. The as-received NP suspension was characterized by a main absorption peak centered at about 528 nm, corresponding to gold NPs’ average size of 40 nm [24], in agreement with the results reported by Huefner et al. [25] for gold NPs in PBS. As for the main absorption peak of the NPs within the cellular medium, it resultingly shifted at 531 nm for the as-prepared NPs-inside-medium suspension and at 533 nm for the suspensions measured 24 h and 48 h after preparation (the suspensions were maintained at 37 °C and 5% CO_2_ until being measured). Such spectral shifts are probably related to the adsorption of medium proteins on the NPs’ surface [25]. Therefore, the absorption spectra ascertain the NPs’ size stabilization when inside the medium. Further, NPs do not aggregate with each other until 48 h after suspension preparation. This is a key step for the design of the NP exposure experiments, because it ensures that the HuKe cells were exposed to NPs, preserving the planned size (40 nm in our case) during the exposure time range. 

### 3.2. Biological Assays

**Figure 2:**
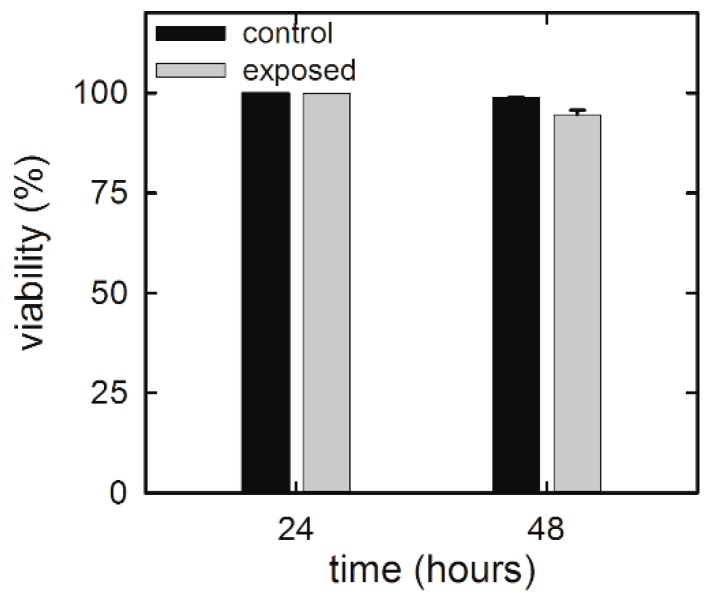
Trypan blue assay for HuKe cells exposed for different times to the gold NP suspension.

Viability assays are a fundamental step in investigating the toxicity of gold NP exposure. In particular, they play a key role in investigating potential side effects of drug-delivering NPs on benign human cells surrounding the tumor cells. In fact, such issues should always be considered in the development of potential anti-cancer therapies. Figure 2 shows the viability results of NP-exposed HuKe cells evaluated by means of a trypan blue assay. Viability results indicate that NP exposure slightly affects the viability in a time-dependent manner. In particular, the viability of exposed cells is unchanged with respect to that of the control ones after 24 h exposure, whereas it is slightly decreased (by about 4%) after 48 h exposure. Overall, the results shown in Figure 2 suggest that the NP exposure, at the investigated concentration, is not cytotoxic for HuKe cells. 

These results are not surprising because it is largely reported that cellular toxicity of gold NPs depends on different parameters, such as particle size [26], shape [27], and concentration [8], as well as cell type [9,10]. As for as human keratinocyte cells, few results have been recently reported. In particular, Li et al. [28] found that different types of coated gold NPs, with size of about 40 nm, are nontoxic for human epidermal keratinocytes (HEK cell line) up to a concentration of 24 µg/mL for 24 and 48 h exposure. In addition, Lu et al. [7] showed that low-size (10–15 nm) gold NPs are barely cytotoxic for human non-malignant keratinocytes (HaCaT cell line) after 72 h exposure. Therefore, our results are in good agreement with those reported in the literature for similar cell lines (keratinocytes).

### 3.3. Raman Spectroscopy

Raman spectra of cells belonging to the same cell type can be different to each other due to the large spatial resolution of Raman microspectroscopy and heterogeneity of the subcellular compartments inside the volume sampled by an exciting laser. Therefore, a proper vibrational analysis to investigate global changes in the cellular structure and composition should be based on the comparison among average Raman spectra instead of single Raman spectra. In order to discriminate information related to different cellular constituents, we measured two Raman spectra for each investigated single cell, by focusing the laser beam firstly on the nucleus and then on the plasmatic membrane outside the nucleus. In the former case, the sampled volume contains, to a greater extent, nucleus components (nucleic acids, DNA/RNA, and proteins) and, to a lesser extent, plasmatic membrane components; in the latter case, the nucleus components were excluded from the sampled volume and the cytoplasm was mainly sampled. 

It is worth remarking that, although the wavelength of the exciting laser (514.5 nm) is within the NP absorption range, the analyzed Raman measurements were not affected by laser–NP interaction, because the laser spot size (about 0.5 µm diameter) was extremely smaller than the cell surface area. Therefore, in most measurements there is no NP within the cellular volume sampled by the exciting laser. On the contrary, only cellular components exposed to NPs are sampled and contribute to the Raman spectrum. However, sometimes a few NPs could be also sampled by the laser beam—in this case, the obtained spectrum is characterized by a large photoluminescence band, as shown in Figure S1 of Supplementary Information, attributable to the excitation of surface plasmons by laser light [29]. When this happened, the corresponding spectrum was discarded (because the subtraction of the background would yield unreliable results) and measurements were repeated by focusing the laser spot on a different cellular area. Such a discard process was related to the fact that the aim of this work was only related to the spectral detection of biochemical modifications induced in cellular components after exposure to a low dose of gold NPs, but not to the spectral detection of NP uptake. 

**Figure 3:**
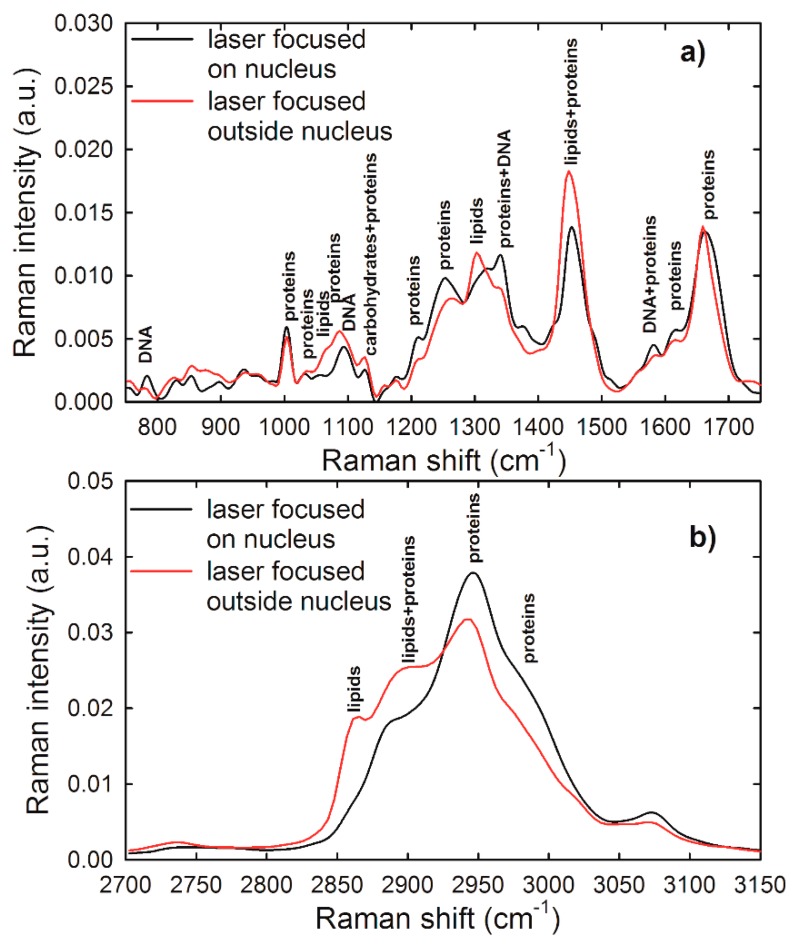
Normalized and averaged Raman spectrum of about 30 unexposed HuKe cells, measured in the low wavenumber (**a**) and high wavenumber (**b**) spectral range, by focusing the exciting laser on nucleus (black lines) and outside nucleus (red lines). The labels refer to the attribution of the most important spectral features reported in [30].

In order to detect spectral markers of specific cellular components, a comparison between average Raman spectra of HuKe cells, measured by focusing the laser beam on the nucleus and outside the nucleus, is reported in Figure 3a,b for the low wavenumber and high wavenumber spectral range, respectively. Such spectra are characterized by many Raman features, which have been assigned according to the published literature [30]; the most prominent ones are labeled in Figure 3. The main spectral differences between these spectra are due to a large contribution of vibrational modes of DNA and nucleic acids to the spectrum sampling nucleus and to a great influence of lipid-related peaks to the spectrum excluding nucleus contribution. The major Raman features of the low wavenumber spectral range in both spectra are related to protein vibrational modes, specifically amide I (at about 1660–1665 cm^−1^), amide III (at about 1250–1270 cm^−1^), CH (at 1340 cm^−1^) and CH_2_ (at 1340, 1450 cm^−1^) bending mode, ring breathing mode of Phenylalanine (at 1003 cm^−1^), and CC (at 1060, 1082, 1580, 1620 cm^−1^) and CN (1060, 1082 cm^−1^) stretching mode. In the spectrum sampling cytoplasm, a well resolved lipid vibrational peak was observed at 1300 cm^−1^ (CH_2_ bending), although the lipid contribution to Raman spectrum was overlapped to the protein signal at 1060, 1450, and 1660 cm^−1^. Instead, a well resolved Raman peak due to nucleic acids and DNA/RNA vibrational modes was centered at 785 cm^−1^ (O–P–O stretching mode of the phosphodiester bond of the phosphate group), whereas other DNA/RNA-related peaks were overlapped to protein peaks in the spectrum sampling nucleus, particularly at 1090 cm^−1^ (PO_2_^−^ phosphodioxy bond of phosphate group), 1340 cm^−1^ (CH bending), and 1378 and 1580 cm^−1^ (ring breathing of DNA/RNA bases). The feature at 1300 cm^−1^ was not resolved in the Raman spectrum sampling nucleus—therefore we can consider as negligible the lipid contribution to such spectrum. The main spectral features in the high wavenumber spectral region were due to the CH_3_ asymmetrical stretching mode of proteins and lipids (2965 cm^−1^), the CH_3_ symmetrical stretching mode of proteins and lipids (2945 cm^−1^), the CH_2_ asymmetrical stretching mode of lipids (2900 cm^−1^), and the CH_2_ symmetrical stretching mode of lipids (2865 cm^−1^). In the spectrum related to cytoplasm, the relative intensity of lipid-related features was quite larger than the corresponding intensity in the spectrum measured on the nucleus, thus confirming that the lipid contribution in the latter spectrum can be considered as negligible. 

**Figure 4:**
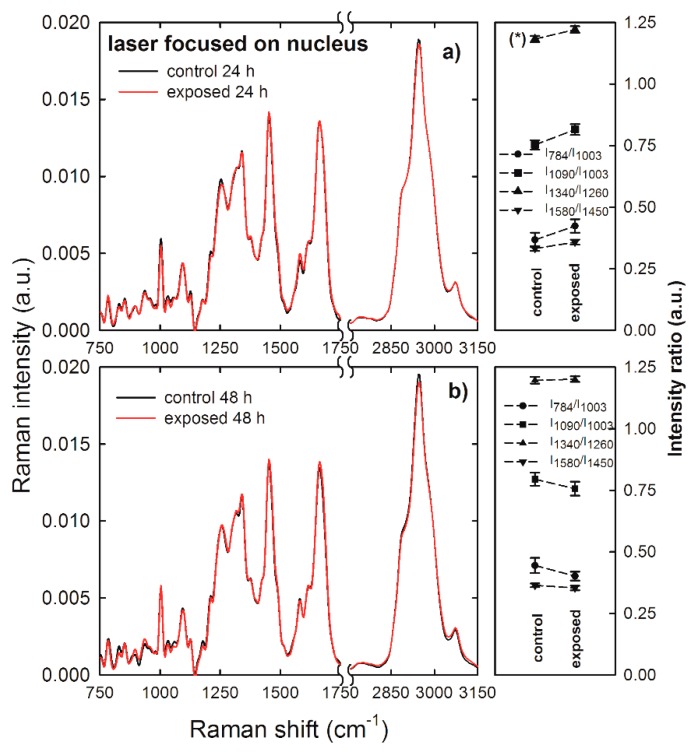
Average Raman spectra of control (black lines) and gold NP-exposed (red lines) HuKe cells, with exposure time of 24 h (**a**) and 48 h (**b**). Each spectrum is the average of spectra collected from about 30 different cells, by focusing the exciting laser beam on the cell nucleus. On the right hand side of each Raman spectrum is shown the corresponding comparison between mean values of the I_784_/I_1003_ (dots), I_1090_/I_1003_ (squares), I_1340_/I_1260_ (triangles up), and I_1580_/I_1450_ (triangles down) intensity ratios of Raman peaks of control and exposed cells. The values are means ± standard error. The values of intensity ratios which are significantly different are labelled (*).

Normalized and averaged Raman spectra measured from the nucleus compartment of control (black lines) and exposed (red lines) HuKe cells are shown in Figure 4a,b as for 24 and 48 h exposure, respectively. Most features of these Raman spectra are very similar, and they are characterized by an overlapping intensity, but few of them show small intensity differences. To better investigate the effects of NP exposure, we compared (for the two time ranges) the ratio of the intensity of the 784 cm^−1^ peak with respect to that of the 1003 cm^−1^ peak (I_784_/I_1003_), as a marker of the DNA-related modification with respect to that of proteins. Similarly, we compared the ratio of the intensity of the 1090 cm^−1^ peak with respect to that of the 1003 cm^−1^ peak (I_1090_/I_1003_), the intensity of the 1340 cm^−1^ peak with respect to that of the 1260 cm^−1^ peak (I_1340_/I_1260_), and the intensity of the 1580 cm^−1^ peak with respect to that of the 1450 cm^−1^ peak (I_1580_/I_1450_). Therefore, the Raman spectra sampling the nucleus region can be used to investigate the influence of NPs on DNA components, by means of the intensity ratio between such components and the protein ones. The values of such intensity ratios are shown at the right hand side of Figure 4a,b for the 24 and 48 h exposure, respectively. Although small differences are visible between the values of the above intensity ratios in the exposed cells with respect to the control ones, none of them were statistically significant. Therefore, we can deduce that the nanoparticle exposure does not significantly modify the density ratio between DNA and protein components in the nucleus region of HuKe cells. These results are in good agreement with results recently reported by other authors. Indeed, Perde-Schrepler et al. [31] reported that HaCaT cells exposed to ~12 nm diameter gold NPs at concentration values of 6 and 15 µg/ml revealed no significant DNA lesions within 48 h exposure time. In addition, by considering cell lines other than keratinocytes, DNA damage was not detected in human liver cancer cell lines (HepG2) exposed to gold NPs with concentrations up to 0.2 μg/mL [32]. Further, it has been reported that gold NPs induced DNA damage in a human lung carcinoma cell line, but such damage is largely repaired over 24 h, indicating that the observed damage is of a transient nature [11].

**Figure 5:**
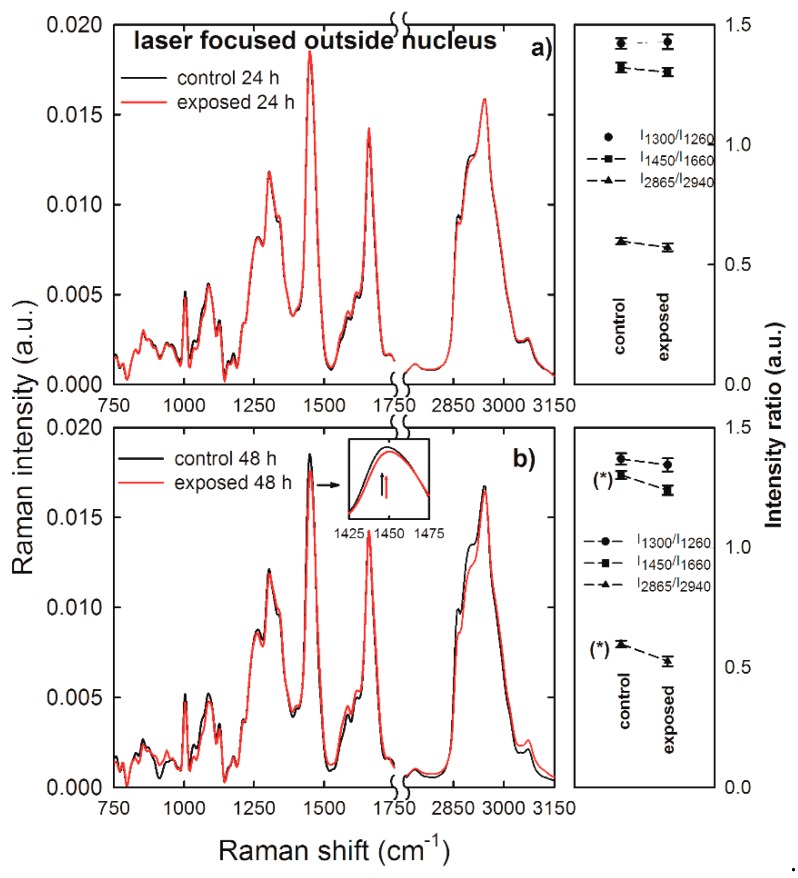
Average Raman spectra of control (black lines) and gold NP-exposed (red lines) HuKe cells, with exposure time of 24 h (**a**) and 48 h (**b**). Each spectrum is the average of spectra collected from about 30 different cells, by focusing the exciting laser beam outside of the cell nucleus. An enlarged detail of the spectra for the 1425–1475 cm^−1^ spectral range is shown in the inset. On the right hand side of each Raman spectrum is shown the corresponding comparison between mean values of the I_1300_/I_1260_ (dots), I_1450_/I_1660_ (squares), and I_2865_/I_2940_ (triangles up) intensity ratios of Raman peaks of control and exposed cells. The values are means ± standard error. The values of intensity ratios which are significantly different are labelled (*).

As lipid-related peaks are weakly resolved in the spectra of Figure 4, we measured Raman spectra by focusing the laser exciting spot outside the nucleus to investigate if NP exposure had damaged lipid bonds of HuKe cells. In fact, Raman spectra sampling membrane and cytoplasmic components allow the discrimination of eventual modifications of the ratio between lipid and protein bond density. Normalized and averaged Raman spectra measured outside the nucleus compartment of control (black lines) and exposed (red lines) HuKe cells are shown in Figure 5a,b as for 24 and 48 h exposure, respectively. Some differences are clearly visible between the intensity values of some spectral features of control spectrum with respect to the corresponding features of exposed spectrum, particularly where the 48 h exposure spectrum is concerned. To compare the effect of NP exposure on lipid bonds with respect to protein ones, we selected and investigated the following intensity ratios: the intensity of the 1300 cm^−1^ peak with respect to that of the 1260 cm^−1^ peak (I_1300_/I_1260_), the intensity of the 1450 cm^−1^ peak with respect to that of the 1660 cm^−1^ peak (I_1450_/I_1660_), and the intensity of the 2865 cm^−1^ peak with respect to that of the 2940 cm^−1^ peak (I_2865_/I_2940_). The values of such intensity ratios are shown at the right hand side of Figure 5a,b for the 24 and 48 h exposure, respectively. Statistically significant differences were visible between the values of the above intensity ratios in the exposed cells with respect to the control ones, particularly where the 48 h exposure time is concerned. Indeed, the intensity ratio I_1450_/I_1660_ has a lower value in the exposed cells than in the control ones; such a decrease indicates that the density of CH_2_ lipid bonds is lower with respect to CH_2_ protein density bonds. Such a statement is also supported by a slight blue-shift (~5 cm^−1^) of the ~1450 cm^−1^ peak in the exposed with respect to the control cells, as highlighted in the inset of Figure 5b, where the black arrow corresponds to the center of the peak for the control cells and the red arrow to that of the exposed cells. In fact, this peak results from the convolution of CH_2_ Raman signals due both to lipid and protein components, with the lipid vibrational mode centered at a lower wavenumber value with respect to the protein mode [30]. Furthermore, the decrease of lipid CH_2_ bonds is remarked by the trend of the I_2865_/I_2940_ intensity ratio, which clearly decreases in the exposed cells with respect to the control ones. Therefore, we can deduce that lipid bonds are much more involved in the NP action than protein ones, mainly for 48 h exposure. This effect is in quite good agreement with the trypan blue assay results, as shown in Figure 2, which were based on the damage of the plasma membrane, where most lipid bonds are located. In addition, the lipid damage could involve also cytoplasmic lipidic structures, such as lysosomes and membrane-bounded cellular compartments, which were sampled by the laser beam. Moreover, the lipid membrane damage as a consequence of gold NP penetration was also asserted in several works based on simulations of interaction of such NPs with model lipid membranes [33,34].

**Figure 6:**
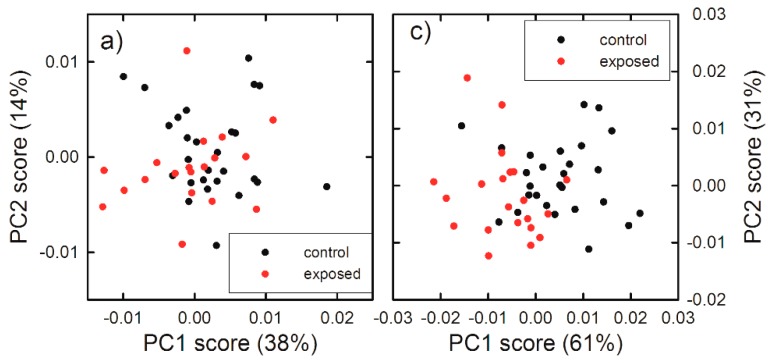

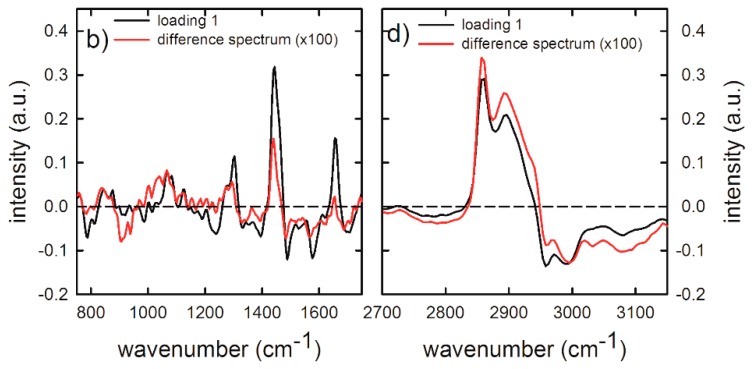
Score plots of the principal component analysis (PCA) of Raman spectra measured in the 700–1800 cm^−1^ (**a**) and 2700–3150 cm^−1^ (**c**) wavenumber range, by focusing the exciting laser beam outside the nucleus. The data refer to the spectra of the 48 h unexposed (black dots) and gold NP exposed (red dots). Loading plots of PC1 (black line) and difference Raman spectra (red line) between 48 h unexposed and exposed cells, measured in the 700–1800 cm^−1^ (**b**) and 2700–3150 cm^−1^ (**d**) wavenumber range. A multiplicative factor has been applied to the difference spectrum to better visualize the comparison with the corresponding loading 1 spectra.

In order to confirm the results of Raman analysis based on intensity ratios, we have performed also a PCA analysis for the spectra measured by focusing the laser beam outside the nucleus, for which significant differences of intensity ratios were observed. In particular, the PCA method is not able to clearly differentiate exposed cells from unexposed ones for 24 h exposure, as shown in Figure S2 of Supplementary Information. Not even the score plot related to the low wavenumber spectral range, shown in Figure 6a, was able to clearly differentiate 48 h exposed cells from 48 h unexposed ones, whereas the score plot related to the high wavenumber spectral range clearly differentiates unexposed and exposed cells, mainly according to the PC1 values, for the 48 h exposure, as shown in the scatter plot in Figure 6c. In particular, the spectra of unexposed cells score positively, whereas spectra corresponding to exposed cells score negatively. The PC1 loadings are reported in Figure 6b,d (black line), for the low and high wavenumber spectra range, respectively. They are compared with the difference signal between average control and exposed spectra (red line). Although the failure of a clear discrimination in the score plot related to low wavenumber spectral range in Figure 6a, the loading 1 plot in Figure 6c can yield some indication about the difference between spectra of exposed and unexposed cells. The strongest positive features of loading 1 in Figure 6b are related to lipid (at 1300 and 1450 cm^−1^) and protein (at 1660 cm^−1^) vibrational modes. In particular, the quite good agreement for spectral position of lipid peaks and the difference spectrum indicates that the main variations in the Raman spectra of exposed and unexposed cells involve the intensity of lipid peaks, especially the peak centered at 1450 cm^−1^, whose intensity ratio with the peak centered at 1660 cm^−1^ is characterized by a statistically significant difference for exposed cells with respect to unexposed ones, as shown in Figure 5. As for the high wavenumber spectral range, the positive features of loading 1 in Figure 6d are related to lipids’ CH_2_ vibrational modes, whereas the strongest negative feature at about 2960 cm^−1^ corresponds to the proteins’ CH_3_ stretching mode. Therefore, unexposed cells were differentiated by exposed ones due to a significant increase of the intensity of lipid signals at 2860 and 2900 cm^−1^ and a decrease of the protein signal at 2960 cm^−1^ in loading 1 spectrum, which was also visible in the difference of the corresponding average spectra. Hence, PCA results are in agreement with those obtained by ratiometric analysis also in the high wavenumber range of 48 h exposed HuKe cells.

## 4. Conclusions

In this study, the nucleus and cytoplasm changes related to molecular events occurring in HuKe cells exposed to gold nanoparticles at doses much lower than the cytotoxic ones have been evaluated for 24 and 48 h exposure, using biological assays and Raman spectroscopy. Significant spectral changes were observed particularly for 48 h exposure, in which a trypan blue assay shows weak viability decrease. Notably, fewer changes were observable in the spectral features of the cell nucleus with respect to those of the cytoplasm, as might be expected, given that the NP uptake occurs through the cytoplasm. In particular, some spectral signatures which are correlated with gold NP exposure have been identified, thus confirming the potential of Raman spectroscopy as a label-free technique to provide multi-parametric information about the biomolecular changes as a consequence of exposure to a low- and non-cytotoxic dose of a stress agent. The most suitable Raman spectral markers to represent the cell response to gold NP exposure were found to be the lipid-related bands at 1450 and 2865 cm^−1^ in the cytoplasm, as demonstrated by ratiometric and principal component analysis. Such Raman spectral markers can potentially be used as biomolecular probes to detect the presence of a very low concentration of novel nanoparticles, provided that a proper investigation of dose-dependence modifications of Raman spectral features is carried out. 

## Supplementary Materials

The following are available online at https://www.mdpi.com/1424-8220/19/10/2418/s1, Figure S1: A typical Raman spectrum obtained in most cases with the laser beam focused outside nucleus (black line), a typical background signal obtained with the laser beam focused outside cell (red line) and a typical Raman spectrum obtained in few cases with the laser beam focused outside nucleus (blue line). In particular, the blue line spectrum, characterized by a large fluorescence background, can be considered an indirect evidence of NPs uptake, because the large fluorescence band can be due to the excitation of surface plasmons of gold NPs as a consequence of the interaction with laser light. Therefore, NPs inside the cell were sampled by the laser beam when such large fluorescence background was observed. Figure S2: Score plots of the PCA of Raman spectra measured in the 700–1800 cm^−1^ (**a**) and 2700–3150 cm^−1^ (**c**) wavenumber range, by focusing the exciting laser beam outside nucleus. The data refer to the spectra of the 24 h unexposed (black dots) and gold NPs exposed (red dots). Loading plots of PC1 (black line) and difference Raman spectra (red line) between 24 h unexposed and exposed cells, measured in the 700–1800 cm^−1^ (**b**) and 2700–3150 cm^−1^ (**d**) wavenumber range. A multiplicative factor has been applied to the difference spectrum to better visualize the comparison with the corresponding loading 1 spectra.
