# Supplementary material for: Biochemical Changes in Human Cells Exposed to Low Concentrations of Gold Nanoparticles Detected by Raman Microspectroscopy
Source: Sensors (Basel). 2019 May 27;19(10):2418. doi: 10.3390/s19102418 (PMC6566781; doi:10.3390/s19102418)
Supplement: Supplementary file 1 [file sensors-19-02418-s001.pdf]

Supplementary Information

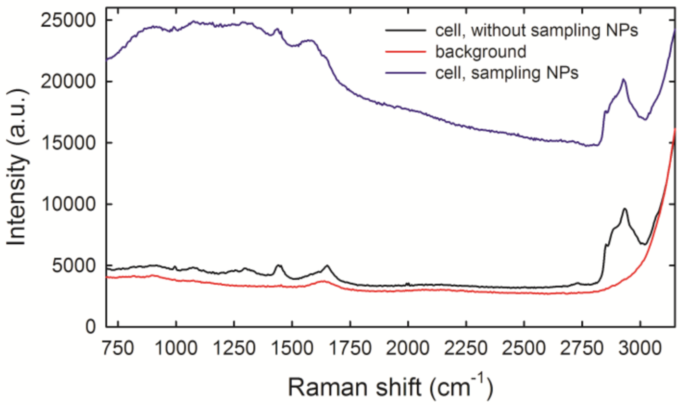

Figure S1. A typical Raman spectrum obtained in most cases with the laser beam focused outside nucleus (black line), a typical background signal obtained with the laser beam focused outside cell (red line) and a typical Raman spectrum obtained in few cases with the laser beam focused outside nucleus (blue line). In particular, the blue line spectrum, characterized by a large fluorescence background, can be considered an indirect evidence of NPs uptake, because the large fluorescence band can be due to the excitation of surface plasmons of gold NPs as a consequence of the interaction with laser light. Therefore, NPs inside the cell were sampled by the laser beam when such large fluorescence background was observed.

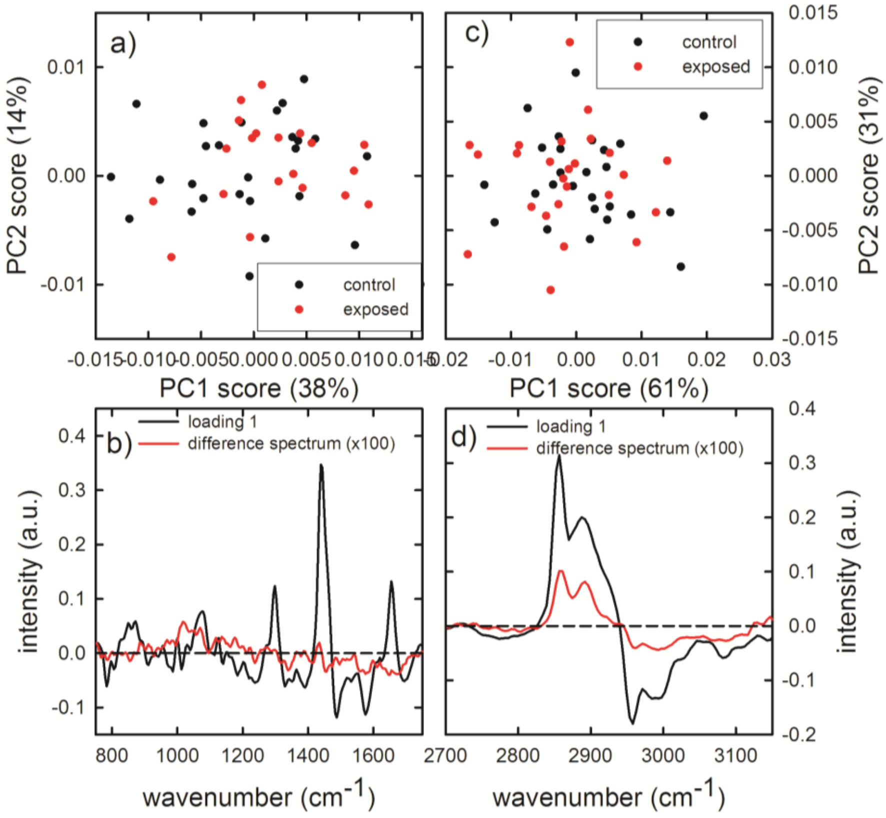

Figure S2. Score plots of the PCA of Raman spectra measured in the 700–1800 cm^{-1} (**a**) and 2700–3150 cm^{-1} (**c**) wavenumber range, by focusing the exciting laser beam outside nucleus. The data refer to the spectra of the 24 hours unexposed (black dots) and gold NPs exposed (red dots). Loading plots of PC1 (black line) and difference Raman spectra (red line) between 24 hours unexposed and exposed cells, measured in the 700–1800 cm^{-1} (**b**) and 2700–3150 cm^{-1} (**d**) wavenumber range. A multiplicative factor has been applied to the difference spectrum to better visualize the comparison with the corresponding loading 1 spectra.

Both the score plots are not able to discriminate exposed cells from unexposed ones. In addition, there is no clear correspondence between spectral features of loading 1 and difference spectrum for the low wavenumber spectral range. On the contrary, a good correspondence occurs for high wavenumber spectral range, although the results of ratiometric analysis do not evidence any statistically significant difference for the peaks intensity ratios. This is due to the small difference values (compare the values of difference spectra in Figure S2d with those in Figure 6d).
